# AI Enabled IoRT Framework for Rodent Activity Monitoring in a False Ceiling Environment

**DOI:** 10.3390/s21165326

**Published:** 2021-08-06

**Authors:** Balakrishnan Ramalingam, Thein Tun, Rajesh Elara Mohan, Braulio Félix Gómez, Ruoxi Cheng, Selvasundari Balakrishnan, Madan Mohan Rayaguru, Abdullah Aamir Hayat

**Affiliations:** 1 Engineering Product Development Pillar, Singapore University of Technology and Design (SUTD), Singapore 487372, Singapore; rajeshelara@sutd.edu.sg (R.E.M.); resor95@gmail.com (B.F.G.); ruoxi_cheng@sutd.edu.sg (R.C.); selvasundarisubramanian@gmail.com (S.B.); mmrayguru87@gmail.com (M.M.R.); abdullahaamir@sutd.edu.sg (A.A.H.); 2 Oceania Robotics, Singapore 627606, Singapore; tun_theinthan@oceaniarobotics.com

**Keywords:** rodent detection, faster RCNN, deep learning, object detection, IoRT, inspection robot

## Abstract

Routine rodent inspection is essential to curbing rat-borne diseases and infrastructure damages within the built environment. Rodents find false ceilings to be a perfect spot to seek shelter and construct their habitats. However, a manual false ceiling inspection for rodents is laborious and risky. This work presents an AI-enabled IoRT framework for rodent activity monitoring inside a false ceiling using an in-house developed robot called “Falcon”. The IoRT serves as a bridge between the users and the robots, through which seamless information sharing takes place. The shared images by the robots are inspected through a Faster RCNN ResNet 101 object detection algorithm, which is used to automatically detect the signs of rodent inside a false ceiling. The efficiency of the rodent activity detection algorithm was tested in a real-world false ceiling environment, and detection accuracy was evaluated with the standard performance metrics. The experimental results indicate that the algorithm detects rodent signs and 3D-printed rodents with a good confidence level.

## 1. Introduction

**Figure 1:**
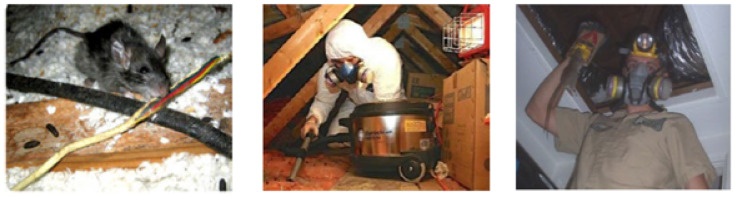
Manual inspection and hazard of pests on false ceiling (source: Google images).

A routine pest control measure is essential in commercial buildings, such as food stalls, offices, shopping malls, etc. Specifically, a routine rodent inspection is mandated in the built environment to prevent rat-borne diseases and to avoid infrastructure damage and electrical or fire accidents. Hence, regular rodent activity monitoring is indispensable to the building management system. Generally, false ceilings seem to be a great place to hide and build their habitat. However, routine rodent inspection in a false ceiling environment has many challenges for pest management companies. Generally, human inspectors are widely used for rodent inspection in a false ceiling environment (Figure 1). They use fluorescent tracking gel, gnawing marks, and droppings to analyze the rodent’s activity. Besides, rodent traps, rodent poison baits, rodent repellents, etc., control rodent infestation. However, the manual inspection method is labor-intensive, time-consuming, and prone to a high risk of accidents because of electrical wire networks, gas pipes, and ducts. Furthermore, due to health difficulties, labor in complex surroundings (maintenance holes, sewage networks), and low wages, a workforce shortage is a key concern for pest management companies. Hence, automating the rodent inspection of the false ceiling is a viable solution.

Robot-assisted inspection has become more popular in the last decade. It is widely used for various inspection applications, including monitoring built environment defects, power transmission line fault detection, ship hull inspection, industry production management, cleaning applications, and so on, as found in the robotics literature [1,2,3,4,5,6,7]. Automating the rodent inspection of the false ceiling using robot-assisted technology is an attractive solution. Recently, a reconfigurable designed wheel for the false-ceiling inspection task was presented in Reference [8], based on the self-reconfigurable robot design using the induction approach discussed in References [9,10]. However, the automation of false ceiling inspection using inspection robots has a lot of practical constraints:

- the systems need an adaptable teleoperated robot with robust locomotion characteristics to operate in dynamic and unstructured complex false ceiling environments;
- the systems need a secure wireless communication framework for controlling and collecting the visual information from the robot; and
- the systems need a rodent activity detection algorithm for automatically detecting rodent signs and rodents from the collected visual feed.

The Internet of Things (IoT) combined with Artificial Intelligence (AI) has been used in various applications in advanced systems, such as transportation, robotics, industrial, and automation systems. The IoRT (Internet of Robotic Things) is a more advanced version of the Internet of Things. It is a combination of many technologies, such as Cloud Computing, Artificial Intelligence (AI), the Internet of Things (IoT), and robotics technology [11,12]. In the IoRT, robots are intelligent machines with sensors that can perceive actions in the actual world by gathering sensor data from numerous sources and taking necessary problem-solving measures. Recently, AI-enabled computer vision has emerged as a technique that can be leveraged to overcome the various shortcomings in robot-assisted remote monitoring and inspection application.

In Reference [13], Gary et al. used robotics and computer vision systems to inspect enclosed spaces in the built environment. The author uses the q-bot inspection robot, mask Regions Convolutional Neural Network (mask-RCNN) to inspect the built environment and reports that the inspection framework detects the enclosed spaces (floorboards, joists, vents, pipes) with 80% accuracy. Muhammad et al. [14] developed a utility pipes structural health monitoring system for a built environment using an autonomous inspection robot and deep learning-based defect detection algorithm. The authors reported that the deep learning algorithm detects cracks in the recorded video inside the pipe with an accuracy of 83.3%. Similarly, the deep learning framework YOLOv3 (You Only Look Once) has been used for the sewer pipe inspection [15]. The authors trained the framework on six types of sewer pipe defects (broken, holes, deposits, cracks, fractures, and roots) and found that the framework detects the defects with an 85.37% mean Average Precision (mAP). A mobile robotic platform with an AI-enabled vision system for real-time construction site monitoring application is proposed in Reference [16]. The robot uses an ENet Pixel-wise semantic segmentation framework for navigating construction sites, monitoring work progress, and structure defect inspection. In References [6,7], authors proposed the shape-shifting robot ’Mantis’ for glass facade crack inspection and cleaned the high rise glass facade building. The robot can automatically move one glass facade panel to another panel without human assistance and also has 15 layers of a deep learning framework for detecting the crack on the glass panel. Quality inspection and assessment robot QuicaBot was reported by Yan et al. [17]. The robot was used for quality assessment of buildings after construction and performed defect identification tasks, including hollowness, alignments, cracks, evenness, and inclination. Aircraft surface inspection using a reconfigurable, teleoperated robot, ’Kiropter’ was reported by Balakrishnan et al. The authors developed a reconfigurable vertical climbing robot for reaching limited places, overlapping joints, and the fuselage of the aircraft’s body. Further, they used deep learning for recognizing and classifying the stains, as well as the defects of an aircraft surface [1].

Similarly, the role of IoT, robotics, and computer vision techniques in various animal and pest monitoring applications has been extensively researched [18,19,20,21]. In Reference [22], Ruilong Chen et al. developed an automated image classification algorithm to identify the wild animal badgers from domestic animals in farm buildings. Two deep learning-based image classification frameworks were used in this experiment. One is a self-trained framework (CNN-1) trained from scratch using a wildlife dataset. The other one is the AlexNet framework fine-tuned with a wildlife dataset. The author describes that the AlexNet framework obtained 98.05% detection accuracy, which is higher than the self-trained framework (accuracy of 95.58%). Another research study by Stefano Giordano et al. [23] reported the creation of an Internet of Things application for crop protection utilizing a monitoring and repelling system to avoid animal invasions and possible damages. In Reference [24], Sabeenian et al. introduced a wild animals intrusion detection system, where a deep learning-based object detection framework is trained for intrusion detection and repelling system to protect the crop against animal attacks. Nikhil et al. [25] suggested a similar system, in which real-time monitoring of agricultural land with crop prediction and animal infiltration avoidance was carried out utilizing the Internet of Things and machine learning for cutting-edge decision-making. Hung Nguyen [26] presented deep convolutional neural network architectures for automating the wildlife monitoring system. The deep neural network architectures are trained to recognize and identify wild animal species automatically. The experiments results indicate that the trained scored 96.6% accurate in recognizing animals and 90.4% accurate in recognizing animal species among the captured images.

IoRT-based rodent inspection is a new approach. It has not been explored yet. This research presents the IoRT framework for rodent activity monitoring in a false ceiling environment. The framework comprises a teleoperated false ceiling inspection robot named Falcon, a remote computing system (local server/cloud server) for executing the deep learning-based rodent activity detection algorithm, and an Ultra-Wideband (UWB)-based indoor localization module for identifying the rodent activity region. This IoRT scheme will help overcome the shortcomings in the existing false ceiling pest control and inspection task in commercial buildings, such as food stalls, offices, shopping malls, etc. Further, routine rodent inspection in the built environment has mitigated rat-borne diseases and avoided infrastructure damage and electrical or fire accidents. The proposed system was developed as per the National Environmental Agency and National Robotics Program Singapore guidelines and tested in various real-time false ceiling testbeds in Singpore.

This manuscript is organized as follows: After providing an introduction, motivation, and literature review in Section 1, Section 2 explains the proposed system. The experimental setup, results, and discussion are demonstrated in Section 3, and Section 4 concludes this research work.

## 2. Overview of Proposed System

**Figure 2:**
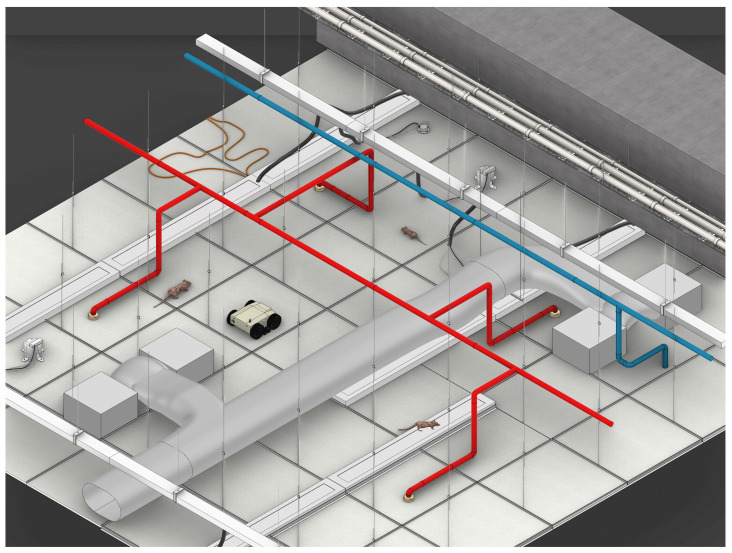
Overview of false ceiling inspection method using the Falcon robot.

**Figure 3:**
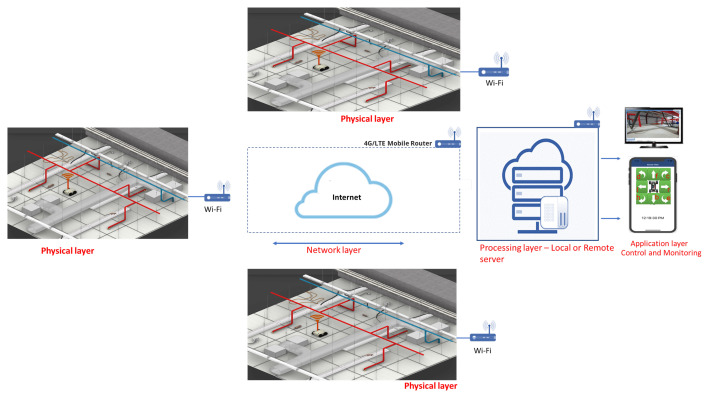
IoRT framework for rodent inspection in a false ceiling.

Figure 2 and Figure 3 show the overview of the proposed system and the AI-enabled IoRT framework. The proposed system uses our in-house developed robot Falcon and a four-layer IoRT framework for detecting rodents in a false ceiling environment.

### 2.1. IoRT Framework

A four-layer IoRT framework [27] is used for rodent inspection in a false ceiling environment. It consists of: (1) a physical layer, (2) a network layer, (3) a processing layer, and (4) an application layer. The details of each layer and its operation are described as follows.

#### Physical Layer

**Figure 4:**
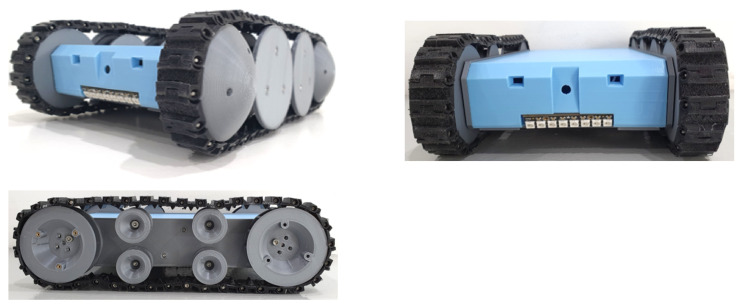
Falcon in multiple perspectives.

In our work, the physical layer is represented by the Falcon robot and its sensors and actuators. Figure 4 shows the overview of our in-house developed false ceiling inspection robot, Falcon. The detailed specifications of Falcon are presented in Table 1. The robot is designed to overcome the various operational constraints imposed by the false ceiling environment. Generally, gypsum board or Plaster of Paris (POP) are two commonly found materials to construct false ceiling panels. However, both are relatively fragile and not robust enough to use as a typical robotic terrain. Moreover, false ceilings are usually congested with piping (sprinkler pipes, air duct pipe, insulation pipe for wires), electrical wiring, suspended cables, and protruding components (top: over-hanged air-conditioning duct pipe, structural beams; and bottom: different sizes of runners and lighting fixtures), and these components obstruct the robot’s operation on the false ceiling. These operational constraints were taken into account and carefully designed for our robot to adapt to the false ceiling environment.

**Figure 5:**
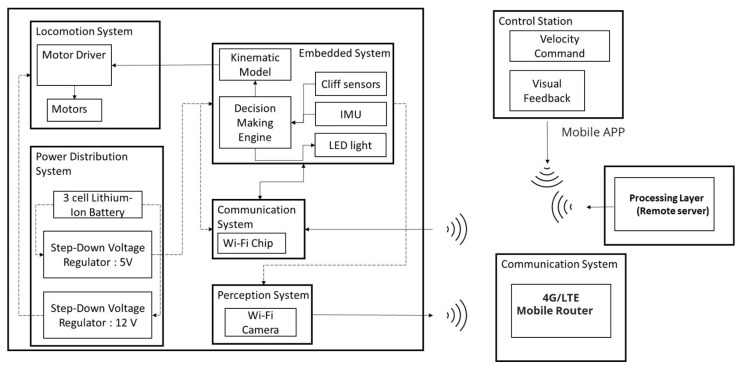
System architecture of Falcon.

Figure 5 illustrates the overall system architecture of Falcon. It consists of the following units: (1) a locomotion module, (2) a control unit, (3) a power distribution module, (4) a wireless communication module, and (5) a perception sensor.

**Locomotion Module:** The locomotion system is directly linked to the overall form factor of the Falcon. It is essential to cover all contact points of the body within the frame of the locomotion system to overcome most of the obstacles in the false ceiling environment. Moreover, it has to span across the entire body to overcome some sharp frames, such as the runners. A typical wheel drive could not operate on the false ceiling as the bottom of the Falcon’s body frame would be exposed to obstacles, resulting in the loss of the contact point of the locomotion system with the terrain. In addition, the width, height, and length of the locomotion system are constrained by commonly found obstacles on the false ceiling. For instance, the current locomotion system is configured to be a track drive of 236 (L) × 156 (W) × 72 (H) (mm × mm × mm) to access narrow spaces with low-hanging obstacles and ground-protruding obstacles of about 55 mm height. Both sides of the locomotion modules are integrated with hemispherical attachments to avoid stabilizing laterally. By integrating them, the Falcon can operate regardless of the direction in which it flips over. Driving motors are chosen with the specifications of a safety factor of 2 to operate continuously on a maximum inclined slope of 12 degrees at the rated torque. The motors can drive the Falcon itself to flip over in the direction of driving against a wall. The motors are chosen with over-specification as the operational terrain of the false ceiling could impose uncertainty on Falcon.

**Control Unit:** The control unit of Falcon was built using a Teensy embedded computing device. The control unit processes the input signals (velocity commands from the user, cliff sensors, IMU) and generates output signals (individual motor speeds using the inverse kinematic model and sensor inputs, and LED lights to support the Perception System). The robot operator sends the velocity command from the control station through the MQTT server. The client-server of the Communication System on Falcon receives the velocity command and transfers it to the embedded control unit. The embedded system control unit computes the velocity commands using the inverse kinematic model to generate individual motor speeds for the motor drivers. Falcon autonomously stops without the operator inputting after triggering cliff sensors to detect an opening in the false ceiling. Cliff sensors and IMU are integrated and calibrated such that the Falcon detects the opening, but it does not falsely decide to stop while overcoming the obstacles due to the increased depth sensed by the cliff sensor. The Power Distribution System regulates 3-cell lithium-ion battery to generate 12V to the motor driver and 5V to Embedded System and other sensors.

**Perception System:** The perception system consists of a Wi-Fi camera, and, through the dedicated router, it transmits the visual feedback to the robot operator at the control station. The visual feedback can be captured as images or videos by the robot operator. This feature helps collect data of rodents, rodent droppings, gnawing marks, and any other information indicating the presence of rodents on the false ceiling.

**UWB Localization Module:** The UWB-based indoor localization technique is used in Falcon for positioning the robot on the false ceiling. In UWB-based localization, the stationary beacons are installed on protruding beams or sidewalls, aiming the antenna at each other, and the mobile beacon is attached to the top of the Falcon. Typically, a minimum of three beacons are necessary to cover the small false ceiling environment, and their count varies according to the size of the false ceiling infrastructure. Through UWB localization, the operator can estimate the absolute location of the Falcon inside the false ceiling. Further, sensor fusion has been adopted to mitigate localization errors and compute an accurate position. It fuses the wheel odometry, IMU data, and UWB localization information and provides a better-estimated location.

### 2.2. Network Layer

The network layer serves as a communication link between the physical layer (robot nodes), processing layer, and application layers. Its functions include providing control communication between the robot and the operator and transferring the collected false ceiling images to the processing layer. D-Link 4G/LTE mobile router was used to establish internet communication. It uses the USIM card to connect to the Internet. Further, the modem has a built-in Wi-Fi router that works under the WPA encryption standard. Finally, the robot captured images are transmitted to a remote server via a 4G/LTE mobile router.

### 2.3. Processing Layer

A local/remote server serves as the processing layer. It includes high-speed computing devices to process high-resolution image and video frames, run the rodent activity detection algorithm, and manage application layer requests.

#### Rodent Activity Detection Module

The Deep Neural Network (DNN)-based object detection framework is used for rodent activity inspection in a false ceiling environment. Generally, the critical inputs to rodent activity detection modules are gnawing, scampering, scratching, droppings, and live detection of rodents in the false ceiling environment. However, detecting these objects in the complex false ceiling environment needs an optimal detection algorithm because these rodent signs are minor, and their pattern is too random. Generally, extracting key detection features from small objects is extremely difficult in any object detection method because there is a high likelihood that small items will become pixelated or overlap, making it impossible to retrieve some usable data. So, inspection algorithms need a small object detection capability.

**Figure 6:**
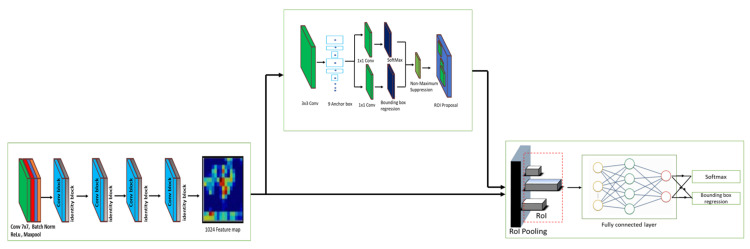
Faster RCNN RestNet architecture.

The Faster RCNN object detection framework was used in the rodent activity detection task, an optimal algorithm for detecting small objects from a complex background. Figure 6 shows the architecture of the Faster RCNN algorithm [28]. It comprises a ResNet 101 (Residual Neural Network) feature extractor, a Region Proposal Network (RPN), a detector, and a classifier head. The framework performs the object detection task in two steps: extracting the feature map and generating the region proposal in the first step, and then identifying the object from each proposal in the second stage. The following sections go over the specifics of each component.

**Feature Extractor:** ResNet 101 is an image classifier algorithm used as a base network for Faster RCNN and performs the feature extraction task. It operates in five phases. The first stage comprises a 7 × 7 convolution kernel, a Batch Normalization (BN) function, the ReLU function, and the max-pooling function. The remaining four phases are composed of residual convolution and identity functions. In this case, the residual conv block is made up of three convolution layers (1×1, 3×3, 1×1) with BN and ReLU, as well as a skip connection with a convolution function of 1×1. Likewise, identity blocks consist of 1 × 1, 3 × 3, and 1 × 1 convolution layers and skip connections. Finally, a 1024-size feature map is generated from the fifth stage convolution and identity layer, fed into the RPN function, detector, and classifier head.

**Region Proposal Network (RPN):** RPN is a critical component of the Faster RCNN detector framework. It generates the bounding box on the output image using the anchor box approach. Anchor boxes are predetermined boundary boxes with a specific height and width. It detects a variety of objects, including objects of varying sizes and overlapping objects. RPN uses the final feature map of the feature extractor as input and performs a 3 × 3 sliding window operation spatially to generate the anchor boxes and a 512-dimensional feature map. The anchor box’s height and width are determined automatically based on the size of the objects in the training dataset. According to the sliding window’s center point, nine anchor boxes are formed for each sliding window region with three different sizes. An anchor box scheme generates the total W×H×k anchors, where W and H are the width and height of the feature extraction layer map, respectively, and k is the depth of the feature map. On the output side, the actual position of an anchor box in the original image is determined by decoding the feature map output back to the input image size using the stride 16 functions. It creates a series of tiled anchor boxes. RPN computes two things from the series of tiled anchor boxes: The first factor is the probability that an anchor is an object. The second thing is to execute the bounding box regression to alter the anchor position to be more appropriate for the objects or more comparable to the ground truth. In the end, the Non-Maximum suppression (NMS) algorithm is applied to filter out the overlapping bounding boxes from the generated proposal.

**Detector and classifier Head:** The detector and classifier head is the final component of the Faster RCNN framework. It composes a Fast RCNN algorithm, Region of Interest (ROI) layer, and an FC layer. The RoI pooling layer takes the RPN-generated proposals and the shared convolutional features from the feature extractor module. For each RPN-generated proposal, the RoI pooling layer extracts a fixed-size feature map. Next, these fixed-size feature maps are sent to the FC layer. Finally, it uses the softmax function and a linear regression algorithm to detect and classify the bounding box of the predicted objects in the image. Further, NMS is applied to improve accurate object localization and eliminate extraneous bounding boxes.

### 2.4. Application Layer

Smartphones and web interfaces are used to carry out the application layer operations. The application layer is used to control the robot from the remote and view the results of the rodent activity detection framework.

## 3. Results and Discussion

This section presents the experimental method and its results. The experiment was performed in two phases. The first phase validates the Falcon robot’s performance in a false ceiling environment in a semi-autonomous mode. In semi-autonomous mode, the navigation control of the robot is performed through a teleoperation mode. The operator has the control to adjust the speed, direction control, and camera focus. During this semi-autonomous mode, the robot automatically avoids obstacles on the false ceiling using a sonar sensor and also pause/stop the robot autonomously when facing obstacles in the false ceiling environment and detect falling regions. The second phase involves validating the rodent activity detection algorithm using rodent signs (gnaw marking, scampering, scratching, droppings) and 3D-printed rodents.

### 3.1. Validation of Falcon Robot Performance

**Figure 7:**
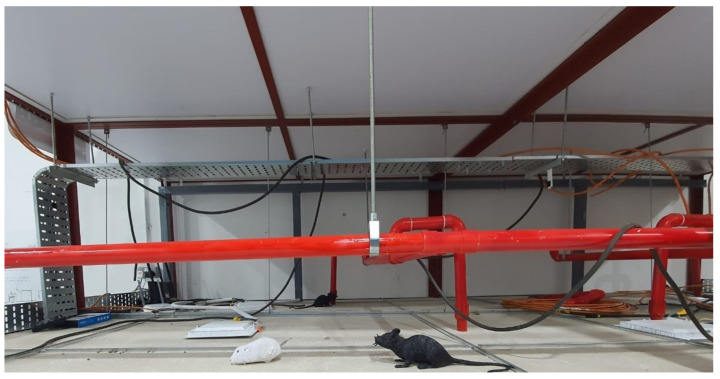
False ceiling prototype test-bed.

**Figure 8:**
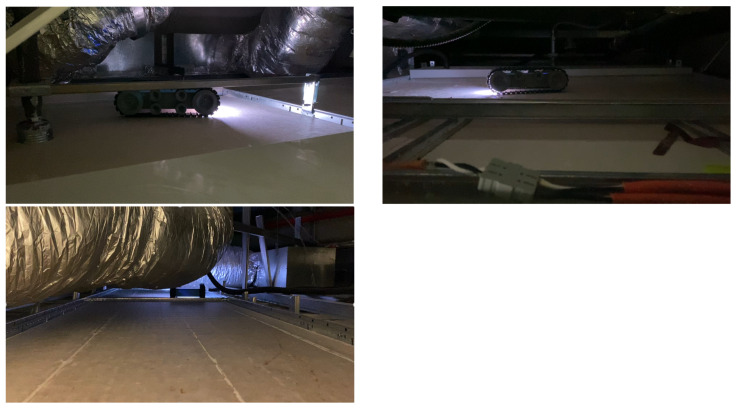
Falcon rodent activity inspection in a false ceiling environment.

The navigation capability of the Falcon robot was tested in prototype false ceiling environments at Oceania Robotics, in Singapore, and an SUTD ROAR laboratory real false ceiling. Figure 7 shows the prototype false ceiling test-bed at Oceania Robotics and in SUTD’s real false ceiling environment. It consists of everyday false ceiling objects, including frames, dividers, pipes, etc. In the ROAR laboratory, the platform was tested in real false ceiling environments (Figure 8), which contained AC vents, electrical and communication cables, etc.

**Figure 9:**
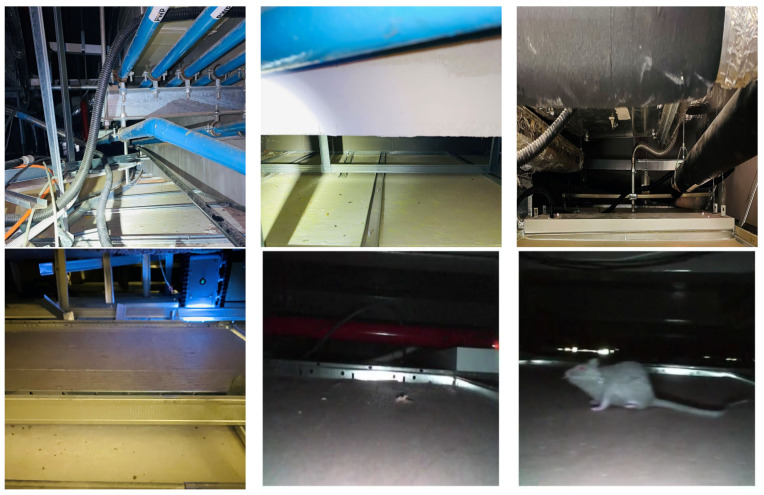
False ceilings environment captured from Falcon robot.

Figure 8 shows the Falcon robot operation in a false ceiling environment. During the inspection, the robot was controlled by 4G LTE-enabled mobile GUI interface and robot positioning was monitored through UWB localization modules fixed in a false ceiling environment. Figure 9 shows some of the Falcon robots collected images with rodent activity signs collected from the SUTD false ceiling. The robot paused in each stage for a few seconds to capture the picture of the false ceiling and forward the remote server via D-Link 4G/LTE mobile router to execute the rodent activity detection.

**Figure 10:**
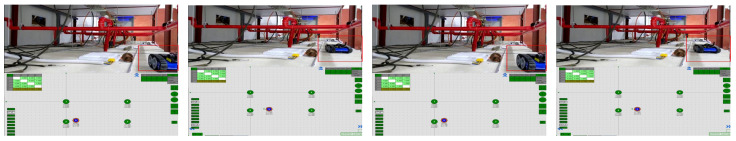
UWB tracking results of the Falcon robot in the Oceania Robotics false ceiling testbed.

**Figure 11:**
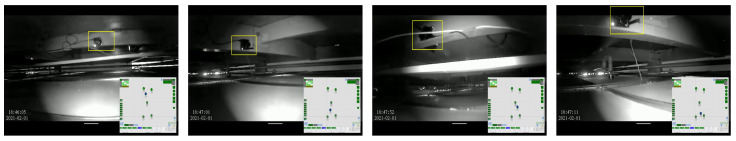
UWB beacon placement in the SUTD false ceilings and tracking results.

The UWB placement and its tracking results are shown in Figure 10 and Figure 11, where one beacon is fixed to the robot and four to five stationary beacons are installed on protruding beams or side walls to obtain optimal tracking and positioning results.

The experiment results show that the developed “Falcon” robot can move around a complex false ceiling environment and is able to accurately capture the false ceiling environment for rodent activity inspection. Further, the UWB localization results (Figure 11) ensure that the Falcon robot can accurately track its position on the false ceiling.

### 3.2. Evaluate the Performance of Rodent Activity Detection Module

**Figure 12:**
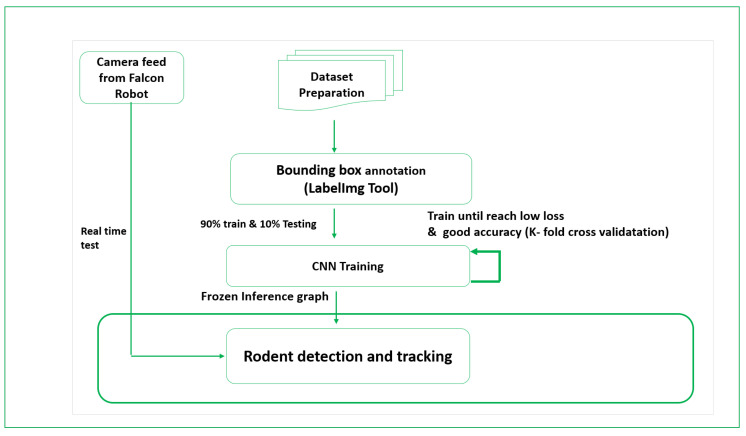
Experimental design flow.

The efficiency of the rodent activity detection algorithm was evaluated in two stages: offline and in real-time. Figure 12 shows the experimental design flow of the rodent activity detection algorithm.

The detection framework has three pre-steps: collecting the dataset (rodent, droppings, and gnaw marks images), labeling the collected images, and training the detection framework with pre-trained weights.

### 3.3. Dataset Preparation and Annotations

The dataset preparation process involves collecting the gnaw marking, scampering, scratching, droppings, and various rodents (Norway rat, roof rat, house mouse). The CNN model was trained and tested using images with a resolution of 640×480. Each class of 1000 images was used in training, collected from various online sources, and in the real environment. Then, image data augmentation (scaling, rotation, and flipping) has been applied to the collected image to mitigate the over-fitting issue and enhance the detection framework learning rate. After the image data augmentation, the dataset was labeled with the “LabelImg” class annotations GUI tool.

### 3.4. Hardware Details

The Tensorflow GPU version 1.9.0 was used to train the detection framework in the transfer learning scheme. The detection framework was trained on an Nvidia GeForce GTX 1080 Ti-powered workstation. The same hardware serves as a remote server and executes the rodent activity detection task.

### 3.5. Training and Hyper Parameter Tuning

In our work, the detection framework was trained using a transfer learning scheme where a pre-trained ResNet 101 algorithm trained on the COCO image dataset was used as a feature extraction module. The framework was trained by Stochastic Gradient Descent (SGD) algorithm and uses the following hyperparameter as optimizer momentum 0.8, initial learning rate: 1 × 10${}^{-}$${}^{2}$, final learning rate: 1 × 10${}^{-}$${}^{5}$, the total number of iterations 70,000, batch size: 5, weight decay was 0.005; decay steps: 10,000. In the RPN training phase, 128 images are randomly selected from the training image database for each iteration and follow the 1:1 ratio of positive (object) to negative (background).

Further, the loss for each prediction is estimated in the training phase as the sum of the location loss $L_{location}$ and confidence loss $L_{confidence}$ (Equation (1)), where confidence loss indicates the prediction error of object class and confidence level. The squared difference between the prediction’s coordinates is referred to as the location loss. To balance the two losses and their impact on the gross loss, a parameter *alpha* (α) is used. Further, the Root Mean Squared (RMS) gradient descent algorithm was applied to mitigate these losses in the training phase.

(1) $$L=\frac{1}{N}\left(L_{confidence}+\alpha L_{location}\right).$$

### 3.6. Dataset Evaluation

The K-fold (K = 10) cross-validation method is applied to the collected dataset to evaluate the training dataset quality. In this method, the dataset is split up into K subsets and K−1 subsets for training, with one subset remaining for performance evaluation. In the training phase, loss and accuracy were estimated for each K-split. In the end, K-fold cross-validation ensures that the images reported are accurate and not biased toward one dataset split over another.

### 3.7. Offline and Real Time Test

**Figure 13:**
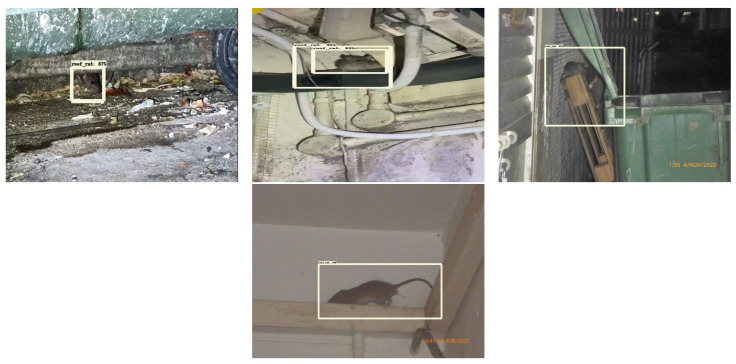
Offline rodent detection test results.

**Figure 14:**
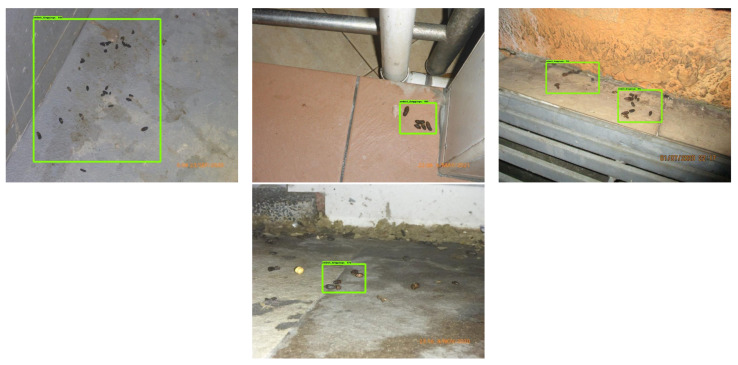
Rodent dropping detection results.

**Figure 15:**
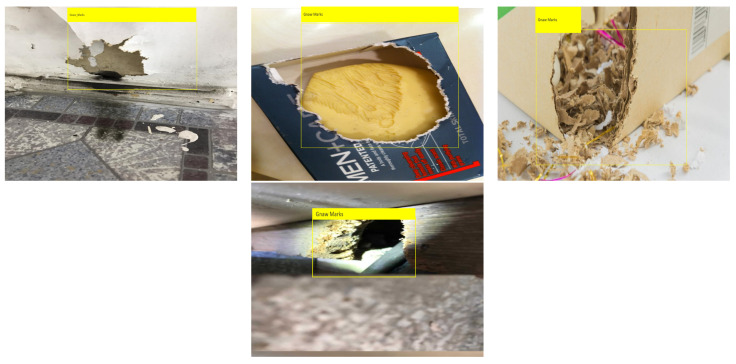
Gnaw marks detection results.

The offline test used rodent sign images (gnaw, scampering, scratching, droppings) and three rodent classes: Norway rats, roof rats, and house mice. One hundred images are used in the offline test for each class. The test images are gathered from various online sources that are not utilized for training the detection framework. Figure 13, Figure 14 and Figure 15 show the experimental results of the offline test. Here, rodent droppings are detected as a gray rectangle box, gnaw marks, scampering, scratching are marked as a yellow rectangle box, and rodent detection is marked as a red, blue, and green rectangle box, respectively. The experimental results show that the algorithm correctly detected gnawing, scampering, scratching, droppings, and three rodent classes, with an average detection confidence level of more than 90%.

Furthermore, the algorithm’s detection efficiency was estimated using standard performance measures (statistical measures), such as accuracy, precision, recall, and $F_{measure}$ [28,29]. Table 2 shows the statistical measures for the offline experimental results. The statistical measures experimental results indicate that the algorithm detected the droppings at an average of 89% accuracy, gnaw marks at 92% accuracy, and rodent class at 94 to 96% classification accuracy.

### 3.8. Evaluate with Prototype Testbed

The real-time rodent activity inspection was evaluated on a prototype testbed, as shown in the Figure 7. For experimental purposes, manually-created gnaw marks, 3D printed rodents, and rodent droppings are placed in various places in a false ceiling testbed. First, the robot was teleoperated in the prototype testbed to identify the rodent signs. Then, the environment was captured with a high-resolution vision system and transferred to the local server to execute the rodent activity detection task.

**Figure 16:**
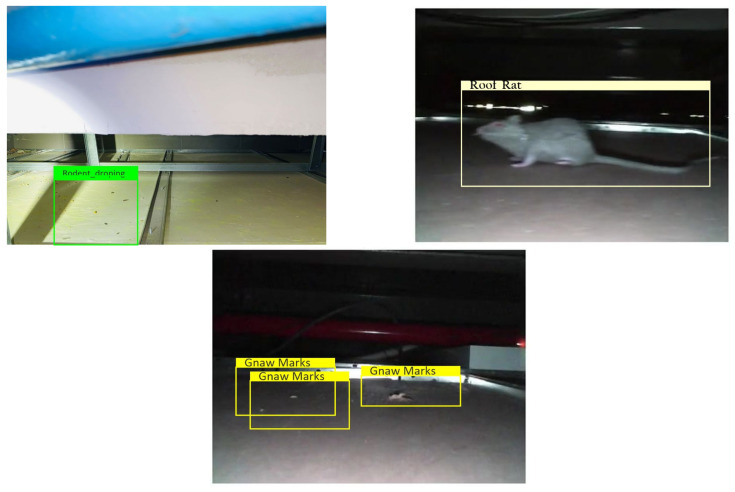
False ceiling testbed detection result.

The experiment was performed with different lighting conditions, rodents with occlusion, and various angles of the rodent’s position. Figure 16 shows the rodent activity detection results in the prototype testbed, and its statistical measures are given in Table 2. The experimental results indicate that the algorithm detects and localizes the rodent signs with 89% accuracy, droppings and 3D printed rodents with an accuracy of 93% confident level, and processes 5 FPS from live video streams.

### 3.9. Experimental Comparisons

Faster RCNN ResNet 101 is compared with YOLOv3 and other Faster RCNN variants, including Faster RCNN Inception and Faster RCNN VGG16. Here, VGG16 (Visual Geometry Group 16) [30,31,32], and the Inception v2 [33] algorithms are employed as feature extractors with Faster RCNN. Likewise, the YOLOv3 module makes use of Darknet-19 as a feature extractor [34,35,36]. The object detection algorithms are trained on the same image dataset and the same number of epochs. The standard performance measures and the processing time of each algorithm were used in the evaluation. The experimental results of three object detection frameworks are reported in Table 3.

The comparison (Table 3) findings show that the Faster RCNN 101 framework outperforms the Faster RCNN VGG16, Faster RCNN Inception v2, and YOLOv3 algorithms in terms of accuracy. In YOLOv3, the miss detection ratio of rodent droppings and rodent’s occlusion was higher than Faster RCNN ResNet 101. Furthermore, for each model, the inference time of each algorithm was estimated. The inference time of each algorithm is given in Table 3. Here, the inference time was calculated by the time taken by the algorithm for executing one image. It is observed that the YOLOv3 framework takes less execution time compared to the Faster RCNN variant. However, the proposed Faster RCNN ResNet 101 ensures the best detection accuracy. Therefore, we can conclude that the proposed Faster RCNN ResNet 101 technique may provide an effective framework for rodent activity tracking in real-time scenarios. The study also found that YOLOv3 has the lowest processing time compared to the Faster RCNN variant. On the other hand, Faster RCNN ResNet 101 achieved the highest detection accuracy than YOLOv3, and the other two Faster RCNN variants include VGG16 and Inception v2. Accurate rodent activity detection is a critical goal in our work. As a result, the Faster RCNN ResNet 101 framework is a better algorithm for rodent activity detection on the false ceiling.

## 4. Conclusions

The rodent activity detection framework in the false ceiling was proposed using the IoRT framework and the in-house developed robot named Falcon. A faster RCNN ResNet 101 object detection framework automatically identified rodent activity in a complex false ceiling environment. The efficiency of rodent activity detection algorithms was tested offline using rodent images, droppings, and gnawing marks, and in real-time with manually-created rodent signs and 3D printed rodents. The performance of the rodent activity detection algorithm was evaluated with standard performance metrics, both offline and in real-time. The experimental results indicate that the rodent activity detection algorithm identifies rodent and rodent marks at an average of approximately 92%. In contrast with other object detection frameworks, including Faster RCNN VGG16, Faster RCNN Inception, and YOLO, the proposed scheme achieved better detection accuracy. The experimental results showed that the trained model had a detection accuracy of 93% and a processing speed of 5 frames per second. The overall experimental results proved that the proposed system is more suitable for automating the rodent activity monitoring task and helping to enhance the inspection service.

## Figures and Tables

**Table 1:** Technical specifications of Falcon.

Description	Specification
Dimensions (L × W × H)	0.236 × 0.156 × 0.072 m
Weight (including battery)	1.3 kg
Type of Locomotion Drive	Track
Top & Bottom Ground Clearance	0.011 m, 0.011 m
Operating Speed	0.1 m/s
Maximum Obstacle Height	0.055 m
Operational Duration	0.5–0.75 h
Battery	3 cells Lithium Ion
Operation Mode	Teleoperation (with integrated sensors to detect fall and stop autonomously)
Communication Mode	Wi-Fi through local MQTT server
Camera Specifications (with on-board Light source)	VGA 640 × 480, up to 30 fps, 60 degree view angle, 20 cm-infinity focusing range

**Table 2:** Statistical measures for rodent activity detection framework.

Class	Precision	Recall	F1	Accuracy
Norway rat	94.58	94.39	94.18	94.57
Roof rat	94.67	94.58	94.26	94.89
House Mouse	95.10	95.05	95.18	94.94
Gnaw markings	92.76	92.54	92.89	92.98
Droppings	89.87	87.23	89.12	89.58

**Table 3:** Comparison analysis.

Algorithm	Precision	Recall	F1	Accuracy	Frames per Second
Faster RCNN VGG16	91.22	90.17	88.22	89.59	6
Faster RCNN Inception v2	93.02	92.58	93.04	92.65	3
YOLOv3	83.46	83.22	81.55	89.33	40
Faster RCNN ResNet 101	93.39	92.78	93.12	93.39	5

## Data Availability

We would like to share the data only on users request.
